# Methylation-Induced Silencing of ALDH2 Facilitates Lung Adenocarcinoma Bone Metastasis by Activating the MAPK Pathway

**DOI:** 10.3389/fonc.2020.01141

**Published:** 2020-07-30

**Authors:** Mengdi Yang, AiTing Wang, Changcan Li, Jing Sun, Gang Yi, Hao Cheng, Xueni Liu, Zhiyu Wang, Yiyi Zhou, Guangyu Yao, Shuai Wang, Rui Liang, Bin Li, Dan Li, Hui Zhao

**Affiliations:** ^1^Department of Internal Oncology, Shanghai Jiao Tong University Affiliated Sixth People's Hospital, Shanghai, China; ^2^Shanghai Institute of Immunology, Shanghai Jiao Tong University School of Medicine, Shanghai, China; ^3^Department of Immunology and Microbiology, Shanghai Jiao Tong University School of Medicine, Shanghai, China; ^4^Department of General Surgery, Tongji Hospital, Tongji University School of Medicine, Shanghai, China

**Keywords:** ALDH2, DNA methylation, lung adenocarcinoma, bone metastasis, MAPK

## Abstract

Bone metastasis (BM) dramatically reduces the quality of life and life expectancy in lung adenocarcinoma (LUAD) patients. There is an urgent need to identify potential biomarkers for application in the treatment of this deadly disease. We compared patient BM, LUAD, and para-LUAD tissues using proteomic analysis and identified aldehyde dehydrogenase 2 (ALDH2), which can detoxify acetaldehyde to acetic acid, as one of the key regulators in lung tumor metastasis. Both the mRNA and protein levels of ALDH2 were significantly lower in tumor tissues than in normal tissues and were lowest in BM tissues with increased migratory capacity. Also, ALDH2 was upregulated following treatment with 5-azacitidine, a DNA methyltransferase inhibitor, in H1299, H460, and HCC827 cells. Further, we identified a potential methylated CpG island 3, with the longest methylated CpG island area in ALDH2, and performed bisulfite genomic sequencing of these sites. An average of 78.18% of the sites may be methylated in CpG island 3. Knockdown of DNA (cytosine-5)-methyltransferase 3A (DNMT3A) and methylated CpG binding protein 4 (MBD4) upregulated ALDH2 expression. ALDH2 functions as a mitogen-activated protein kinase (MAPK) upstream to inhibit cell proliferation and migration, promote cell apoptosis, and alter the epithelial–mesenchymal transition (EMT) by elevating E-cadherin and attenuating vimentin. Cell proliferation and migration were inhibited after the addition of the JNK inhibitor SP600125. In the multivariate analysis, M stage (*p* = 0.003), ALDH2 (*p* = 0.008), and phospho-c-Jun N-terminal kinase (p-JNK) (*p* = 0.027) expression were independent prognostic factors for overall survival in patients with BM. *In vivo* experiments also showed that ALDH2 expression could suppress tumor formation. In summary, we found that ALDH2 expression is a prognostic factor for BM in LUAD and that DNMT3A and MBD4 repression of ALDH2 via a MAPK-dependent pathway alters the EMT process, indicating that these proteins could act as potential biomarkers or therapeutic targets for LUAD metastasis.

## Introduction

Lung cancer is the most lethal malignant disease (1, 2). Delays in the diagnosis of lung cancer progressively worsen the patients' prognosis (3). Most (85%) cases of lung cancer are classified as non-small-cell lung cancers (NSCLC), these malignancies include lung adenocarcinoma (LUAD), which is the most common type of NSCLC. Bone is a common metastatic site for lung cancer patients (4–6). About 30–40% of advanced NSCLC patients will present with bone metastasis (BM), which can induce skeletal-related events (SREs) (7). Once BM occurs, ~80% of patients suffer significant pain, reduced quality of life (8), and increased economic costs (9). Diagnosis of this disease is usually delayed because patients have mild or in some cases no symptoms in the early stages of BM until the first SRE is reported. At this point, the prognosis is poor (10). Large-scale clinical studies have shown that BM is a key feature in patients with poor survival (11). Non-BM patients have significantly longer overall survival (OS) rates when compared to BM patients (10.2 vs. 5.8 months, *p* < 0.05) (12).

Although primary bone tumors and BM tumors have been investigated in many studies, the molecular changes and indicators that could be used to predict BM remain largely unknown in LUAD. Aldehyde dehydrogenase 2 (ALDH2), which is easily detected in a clinical setting, is a multifunctional enzyme involved in metabolic processes and oxidative stress and has been associated with a variety of diseases (13). In the rate-limiting step of ethanol metabolism, ALDH2 catalyzes the conversion of acetaldehyde to acetic acid and has been closely related to esophageal cancer (14), hepatocellular carcinoma (15), and various other cancers (16, 17). Li et al. used cell lines to show that ALDH2 inhibition promoted lung cancer by increasing DNA damage and acetaldehyde accumulation (18). However, the potential role of ALDH2 in the pathogenesis of LUAD and its underlying metastatic mechanism remain largely unknown.

Our results show that ALDH2 expression was lower in LUAD tissues than in their paired normal tissue counterparts and was lowest in LUAD BM tissues at both the transcriptional and translational levels. This is the first time a study has demonstrated a negative correlation between ALDH2 expression and malignant LUAD traits in independent LUAD and BM cohorts. Further analysis showed that DNA methylation plays an important role in ALDH2 expression, which was mediated by DNA (cytosine-5)-methyltransferase 3A (DNMT3A) and methylated CpG binding protein 4 (MBD4), and that the expression of these regulators greatly increased the probability of BM in lung cancer patients. Besides, ALDH2 was shown to inhibit cell proliferation and migration, promote apoptosis, and alter the epithelial–mesenchymal transition (EMT) process. Elevated expression levels of vimentin and phospho-c-Jun N-terminal kinase (p-JNK) and reduced expression of E-cadherin were found in BM patients (*p* < 0.05), and both cell proliferation and migration were inhibited after the addition of the JNK inhibitor SP600125. In the multivariate analysis, M stage (*p* = 0.003), ALDH2 expression (*p* = 0.008), and p-JNK (*p* = 0.027) expression were all shown to be independent prognostic factors for the OS of BM patients. These findings support the hypothesis that ALDH2 influences metastasis largely through the modulation of the mitogen-activated protein kinase (MAPK) signal pathway, especially the activation of p-JNK, which provides a potential target for future therapeutic interventions.

## Materials and Methods

### Patients

From May 2015 to January 2018, 12 LUAD patients, who underwent surgical operations, and 45 LUAD BM patients, who underwent bone biopsies, admitted to Shanghai Jiao Tong University Affiliated Sixth People's Hospital, were enrolled in this study. All patients provided informed consent for their participation. Lung cancer classification was based on the 2015 version (19). All patients were followed up for more than 1 year. Criteria for enrollment included no other tumors, pathology (lung cancer), sample availability, and follow-up. The main relevant characteristics of The Cancer Genome Atlas (TCGA) was previously described in the study (20).

### Proteomic Analysis Using Two-Dimensional Gel Electrophoresis (2-DE)

This experiment comprised multiple steps including protein extraction, sample homogenization, protein quantification, and 2-DE. In protein extraction, tissues were washed with precooled single-strength phosphate-buffered saline (1 × PBS) and cut into 1-mm^3^ pieces (~300 mg each). These samples were ground in liquid nitrogen and then lysed in 1 ml lysis buffer [2 mol/L thiourea, 4 mol/L urea, 0.2% carrier ampholyte (3–10 NL), 4% CHAPS, and cocktail (1:100, Roche)]. Then samples were homogenized using a 4°C Dounce homogenizer, transferred to a precooled centrifuge tube, and then sonicated (80 W, 10 s eight times, 15 s apart, then placed on ice). The whole process was carried out at 4°C or in an ice bath. Lysates were centrifuged at 18,407 g for 1 h, and then the supernatant was collected. Bio-Rad protein assay reagents were used to quantify the protein concentration of each sample, which were then divided into fractions of 100 μg of protein in individual 500-μl centrifuge tubes, and frozen at −80°C. We then loaded these 100 μg protein samples into each lane of a 2-DE gel, using IEF of pH 3–10 on non-linear strips (Amersham). 2-DE was then run at 30 V for 12 h, 500 V for 1 h, 1,000 V for 1 h, 8,000 V for 8 h, and 500 V 4 h. Finally, the gels were dyed using silver staining, scanned on a flatbed scanner, and analyzed using Adobe Photoshop. Next, protein spots that were judged to be differentially expressed between para-LUAD, LUAD primary tumors, and BM were then cut from the gel. These spots were then digested and analyzed by matrix-assisted laser desorption ionization–time of flight **(**MALDI-TOF). Statistically significant protein spots (*p* < 0.05) that exhibited at least a 1.3-fold change were considered differentially expressed.

### Immunohistochemical Assay

Paraffin-embedded samples were incubated with antibodies against ALDH2 (Abcam #ab108306, USA), p-JNK (CST, #4668, USA), vimentin (CST, #5741, USA), and E-cadherin (CST, #3195, USA); washed; and then incubated with biotinylated secondary antibody (Kirkegaard & Perry Laboratories, USA). Fromowitz's criterion was used for semiquantitative assessment of these samples (21). The assessment was completed by two pathologists who were double-blinded to the patient's information.

### Real-Time PCR (RT-PCR)

RNA was extracted using TRIzol reagent (Sigma-Aldrich, USA) and then converted to cDNA using the PrimeScript RT Reagent Kit (TaKaRa Bio, Japan). SYBR Premix Ex Taq (TaKaRa Bio) was used to complete the RT-PCR on a 7900HT Fast Real-Time PCR System (Applied Biosystems, USA). The conditions included 40 cycles of PCR (95°C for 5 s and 60°C for 30 s) after an initial denaturation. β-Actin was used as a control to determine the expression level of each gene. The primers used in this study are listed in Table 1.

**Table 1:** Primers used in the study.

**Gene**	**Sequence**
TET1-forward	GCTATACACAGAGCTCACAG
TET1-reverse	GCCAAAAGAGAATGAAGCTCC
TET2-forward	CTTTCCTCCCTGGAGAACAGCTC
TET2-reverse	TGCTGGGACTGCTGCATGACT
TET3-forward	GTTCCTGGAGCATGTACTTC
TET3-reverse	CTTCCTCTTTGGGATTGTCC
Actin-forward:	GGACTTCGAGCAAGAGATGG
Actin-reverse:	GCACTGTGTTGGCGTACAG
MBD1-forward	CCGAGGGATGAGACCAAGG
MBD1-reverse	GCTCGACAGTCTTTGCACAAC
MBD2-forward	GCAAGCCTCAGTTGGCAAG
MBD2-reverse	ATCGTTTCGCAGTCTCTGTTT
MBD4-forward	CAGGAACAGAATGCCGTAAGT
MBD4-reverse	CCTTGTGGGCTGATAAAGTACAC
DNMT1-forward	AGGCGGCTCAAAGATTTGGAA
DNMT1-reverse	GCAGAAATTCGTGCAAGAGATTC
DNMT3a-forward	TTACACAGAAGCATATCCAGG
DNMT3a-reverse	GAGGCGGTAGAACTCAAAG
DNMT3b-forward	AGGGAAGACTCGATCCTCGTC
DNMT3b-reverse	GTGTGTAGCTTAGCAGACTGG
ALDH2-forward	AACAATTCCACGTACGGGCT
ALDH2-reverse	CTCCCCGACATCTTGTAGCC
shALDH2-A forward	CCGGGCAGATCATTCCGTGGAATTTCTCGAGAAATTCCACGGAATGATCTGCTTTTTG
shALDH2-A reverse	AATTCAAAAAGCAGATCATTCCGTGGAATTTCTCGAGAAATTCCACGGAATGATCTGC
shALDH2-C forward	CCGGGCTGATAAGTACCACGGGAAACTCGAGTTTCCCGTGGTACTTATCAGCTTTTTG
shALDH2-C reverse	AATTCAAAAAGCTGATAAGTACCACGGGAAACTCGAGTTTCCCGTGGTACTTATCAGC

### Western Blot

BM, LUAD, and para-LUAD tissues or total cell lysates were placed in RIPA buffer. An equal amount of protein was added and separated by electrophoresis. The resolved proteins were transferred to a polyvinylidene fluoride membrane, and antibodies against ALDH2 (Abcam #ab108306, USA), EMT markers [Cell Signaling Technology (CST, USA): N-cadherin #13116, E-cadherin #3195, SNAIL #3879, and Vimentin #5741; ZEB1 Proteintech, China: #21544-1-AP], and MAPK and STAT pathway molecules [p-P38 #4511, p-ERK #4370, p-c-Jun #3270, p-JNK #4668, P38 #8690, ERK #4695, and c-Jun #9165 (CST, USA); JNK (Abcam #ab179461, USA); and DNMT3A #sc-373905, MBD4 #sc-365974, BCL2 #sc-7382, and BAX #sc-7480 (Santa Cruz, USA)] were all used as appropriate to evaluate expression. β-Actin (Proteintech #MAB1420, China) was used as the loading control for these experiments. A horseradish peroxidase-conjugated secondary antibody (Jackson ImmunoResearch, USA) was used for visualization. ECL Plus reagents (Millipore, USA) were used to develop the blots, which were then imaged and quantified using ImageJ (1.52 version) software.

### TCGA Data and Kaplan–Meier Survival Analysis

Clinical and expression data can be downloaded from the TCGA datasets. “DESeq” and “survival” packages were used to analyze R. Logrank *p*-values and hazard ratios with 95% confidence intervals (CIs) were calculated using the Kaplan–Meier plotter (http://kmplot.com).

### Cell Culture

Four LUAD cell lines (A549, H1299, H460, and HCC827) and the normal human bronchial epithelial HBE135-E6E7(HBE) cell line were purchased from the Chinese Academy of Sciences Cell Bank. RPMI 1640 was used to culture the HCC827, H1299, and H460 cells, while the A549 and HBE cells were cultured in DMEM (Invitrogen) supplemented with 1% penicillin/streptomycin and 10% fetal bovine serum and grown in 5% CO_2_ at 37°C.

### Transfection

Cells that had grown to a density of 70–80% in six-well plates were transfected. The transfection mixture contained 100 μl Opti-MEM, 12 μl polyethylenimine (PEI), and 4 μg of each of the relevant plasmids. The mixture was allowed to stand for 10 min. To wash the cells, 1 × PBS was used; then Opti-MEM was added (900 μl) to each well, and after that, the transfection mixture was added. The medium was changed 4–6 h after transfection, and cells were harvested 36 to 48 h after transfection.

### Drug Treatment

When cells grew to 80%, they were treated with 5-azacitidine (5-Aza) (Sigma-Aldrich, USA) at a concentration of 0, 0.5, 1, 2, 5, or 10 μmol/ml. SP600125 (20 μmol/ml) was used to inhibit JNK.

### Bisulfite Sequencing PCR

Genomic DNA was extracted from H1299 cells using a ZR Genomic DNA-Tissue MiniPrep Kit (Zymo Research, USA). An EZ DNA Methylation-Gold Kit (Zymo Research) was then used to complete the bisulfite modification of the DNA templates, this bisulfite-modified genomic DNA was amplified, and the PCR products were cloned into the pEASY-Blunt Cloning Vector (Transgene Biotech). The sequencing results were compared with the promoter sequence. The CpG remained unchanged as methylated and changed as unmethylated.

### Lentivirus Packaging

When HEK293T cells grew to a density of 70–80% in 10-cm dishes, cells were transfected with the lentiviral packaging mixture. The transfection mixture contained 600 μl Opti-MEM, 72 μl PEI, and 24 μg of the relevant plasmids, which included 12 μg of the target plasmids, 10.68 μg dR8.9, and 1.32 μg VSV-G. The mixture was allowed to stand for 10 min. Then PBS was used to wash cells. Opti-MEM was added (5.4 ml) to the cells, and after 10 min, the transfection mix was dispensed into the corresponding dishes. The medium was changed 4–6 h after transfection, and the supernatant was collected at 48 and 72 h after transfection.

### Cell Migration and Invasion Assays

We used Transwell chambers (Corning Incorporated, USA) to assess cell migration and invasion. Cells were seeded on top of the membrane in the upper chamber. After incubation for 24 h, cells in the upper chamber were removed. Cells on the membrane surface in the lower chamber were fixed and stained using a 0.5% crystal violet solution. Each chamber was quantified using five random fields.

### Cell Proliferation Assay

Cell Count Kit-8 (CCK-8, Dojindo, Japan) was used to evaluate cell proliferation. The cells were cultured with 3,000 cells in each well of a 96-well plate. We incubated cells for 0, 24, 48, 72, and 96 h. At each time point, CCK-8 was added (10% of the medium volume) and incubated for 2 h at 37°C. One hundred microliters of CCK-8 was transferred to the wells, and the absorbance was measured at 450 nm using a microplate reader (Bio-Rad, USA). The experiments were carried out six times.

### Apoptosis Assay

After transfection (48 h), the cells were harvested, and apoptosis was assessed by flow cytometry using an Annexin V Apoptosis Detection Kit (eBioscience, USA). All samples were run on a BD LSRFortessa (BD Biosciences, USA), and FlowJo software (TreeStar, USA) was used to analyze the data.

### Animal Experiments

Experimental animals were ordered through the Animal Ethics Committee of the Shanghai Jiao Tong University Affiliated Sixth People's Hospital, and all animal experiments were performed using a protocol approved by the Committee. Two million viable PLVX-Flag H1299 or PLVX-ALDH2 H1299 cells were injected subcutaneously into nude male mice (6 weeks). Mouse weight (g), tumor volume (mm^3^), and tumor weight (g) were calculated for each group weekly. Mice were sacrificed when the tumor volume reached 1.2 cm^3^ [*V* = (length × width^2^)/2].

### Statistical Analyses

Comparisons between groups were analyzed using SPSS version 20.0 (SPSS Inc., USA) software. Comparisons were completed using a one-way analysis of variance (ANOVA), two-tailed Student's *t*-test, non-parametric tests, chi-square test, or Fisher's exact test. Kaplan–Meier analyses with logrank tests and the Cox proportional hazard model were used to investigate the hazard ratio and 95% CIs for OS. All data are presented as the mean ± SD. A *p* < 0.05 was considered statistically significant.

## Results

### ALDH2 Expression Is Significantly Negatively Correlated With Cell Survival and BM in Human Patients

**Figure 1:**
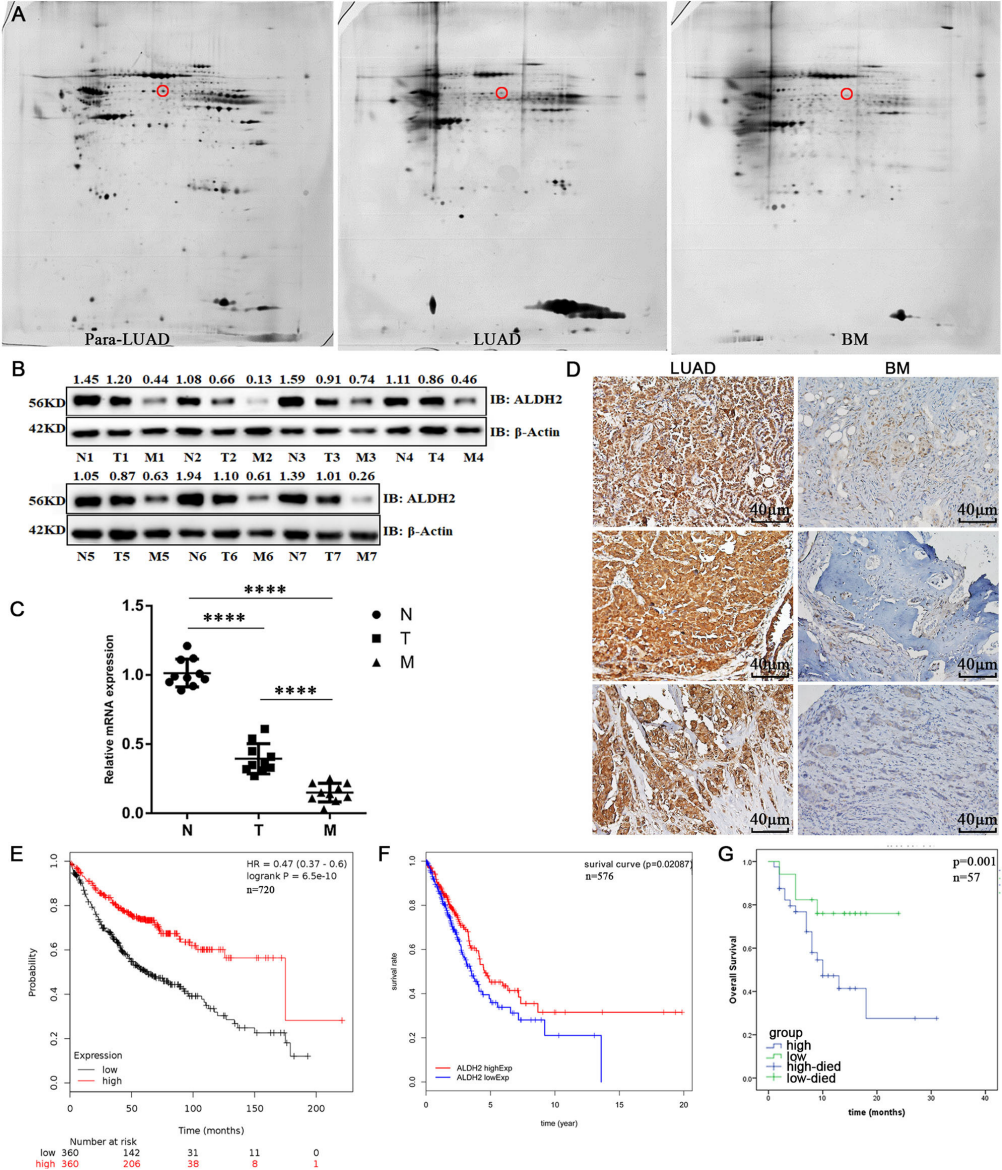
ALDH2 expression is significantly negatively correlated with cell survival and BM in human patients. **(A)** 2-DE was used to identify differentially expressed proteins in para-LUAD, LUAD, and LUAD with BM tissues. The circles denote ALDH2. The expression levels of ALDH2 in para-LUAD, LUAD, and LUAD with BM were all confirmed by western blot **(B)** and RT-PCR **(C)**. IHC was done to assess the expression of ALDH2 in LUAD and LUAD with BM **(D)**. Reduced expression of ALDH2 was significantly associated with poor survival in Kaplan–Meier analysis **(E)** and in TCGA data **(F)**. **(G)** High expression of ALDH2 was associated with good overall survival in LUAD (*n* = 12) and BM (*n* = 45). Three independent samples are shown in the figure. ****p < 0.0001 and *p* < 0.05 were considered significant.

To explore the latent biomarkers of LUAD with BM, we identified between 1,300 and 1,800 spots from 2-DE, which may be differentially expressed among para-LUAD, LUAD, and BM tissues (Figure 1A). ALDH2 appeared to be significantly negatively associated with BM (*p* < 0.05, ratio ≥1.5). To further verify the expression of ALDH2 in tissues, the ALDH2 expression level was further characterized in 57 clinical patients. ALDH2 expression was lowest in BM, followed by LUAD and para-LUAD samples when evaluated by western blot (Figure 1B), RT-PCR (Figure 1C), and immunohistochemistry (IHC, Figure 1D). IHC showed that low expression of ALDH2 was associated with a high risk for BM (*p* < 0.0001; Table 2). We checked whether the expression of ALDH2 was related to reduced OS using Kaplan–Meier plots (Figure 1E), TCGA (Figure 1F), and enrolled patients who were followed-up for more than a year (Figure 1G). ALDH2 expression was significantly correlated with various clinicopathological factors, including smoking; American Joint Committee on Cancer stage (AJCC); T stage, N stage, and M stage; and p-JNK, E-cadherin, and Vimentin expression levels (Table 3).

**Table 2:** Expression of ALDH2 protein in LUAD and BM tissues.

**Tissue sample**	***n***	**ALDH2 expression**		***P*-value**
		**Low (%)**	**High (%)**	
LUAD	12	2 (16.7)	10 (83.3)	<0.0001*
BM	45	36 (80.0)	9 (20.0)	

**Table 3:** Correlation between ALDH2 expression and clinicopathological features.

**Clinicopathological features**	**Total (*n* = 57)**	**ALDH2 protein expression**		***P*-value**
		**Low (*n* = 38) (%)**	**High (*n* = 19) (%)**	
**Age, years**				
<65	37	22 (57.9)	15 (78.9)	0.148
≥65	20	16 (42.1)	4 (21.1)	
**Gender**				
Male	37	24 (63.2)	13 (68.4)	0.775
Female	20	14 (36.8)	6 (31.6)	
**Smoker**				
Yes	9	3 (7.9)	6 (31.6)	0.048*
No	48	35 (92.1)	13 (68.4)	
**T stage**				
T1 + T2	31	17 (44.7)	14 (73.7)	0.051
T3 + T4	26	21 (55.3)	5 (26.3)	
**N stage**				
N0	32	19 (50.0)	13 (68.4)	0.26
N1	25	19 (50.0)	6 (31.6)	
**M stage**				
M0	15	3 (7.9)	12 (63.2)	<0.001*
M1	42	35 (92.1)	7 (36.8)	
**AJCC stage**				
I + II	9	1 (2.6)	8 (42.1)	<0.001*
III + IV	48	37 (97.4)	11 (57.9)	
**p-JNK**				
Positive	23	11 (28.9)	12 (63.2)	0.021*
Negative	34	27 (71.1)	7 (26.8)	
**E-cadherin**				
High	38	29 (76.3)	9 (47.4)	0.039*
Low	19	9 (23.7)	10 (52.6)	
**Vimentin**				
High	22	11 (28.9)	11 (57.9)	0.046*
Low	35	27 (71.1)	11 (42.1)	

* *p < 0.05 indicates a significant difference*.

### The ALDH2 Locus From H1299 Cells Exhibits High Levels of DNA Methylation

**Figure 2:**
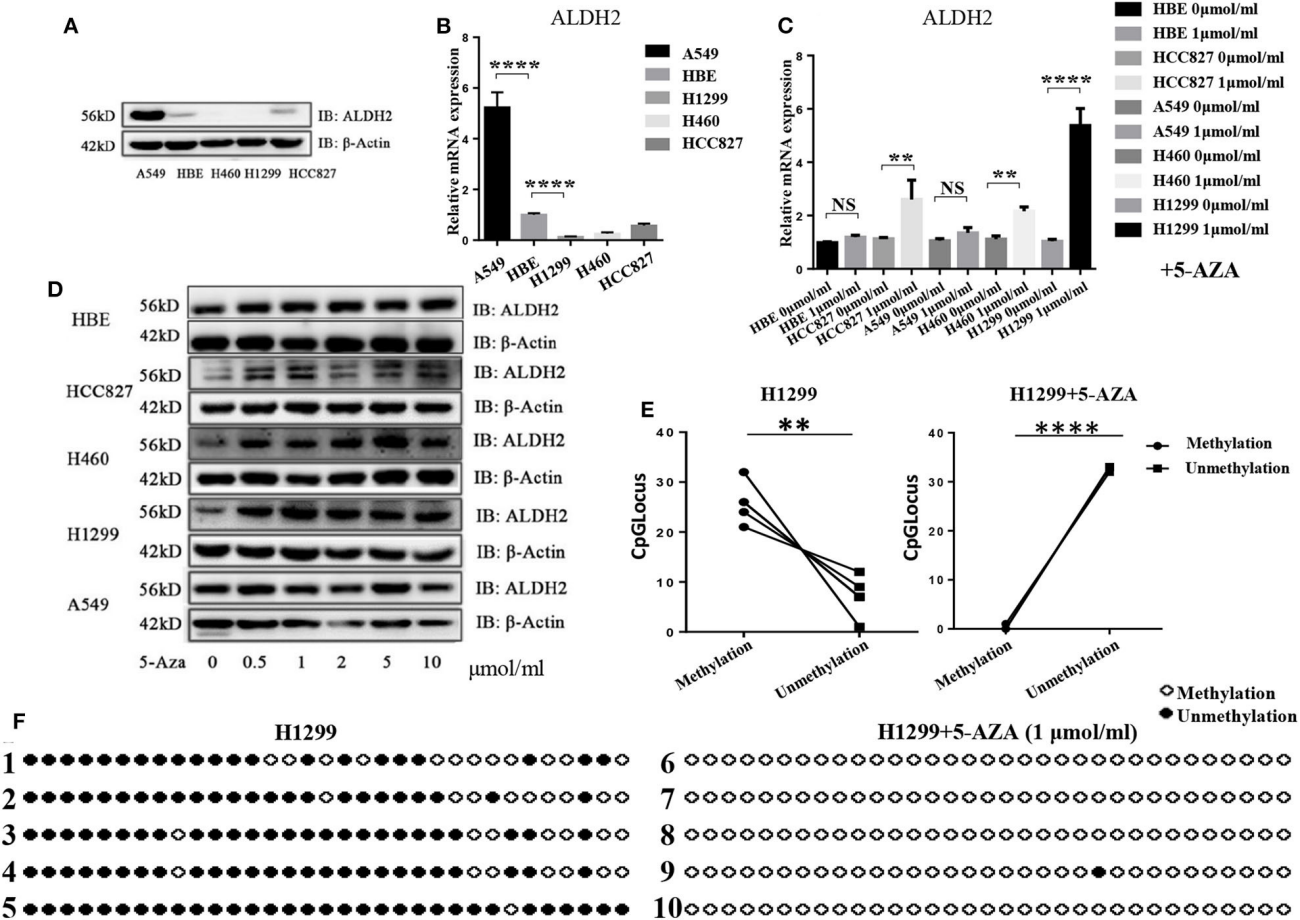
The ALDH2 locus in H1299 cells has high levels of DNA methylation. ALDH2 expression was evaluated in different LUAD cell lines by western blot **(A)** and RT-PCR **(B)**. ALDH2 levels were evaluated after treatment with 0, 0.5, 1, 2, 5, and 10 nmol/ml 5-Aza by western blot **(C)** and RT-PCR **(D)**. **(E,F)** ALDH2 DNA methylation sites in H1299 cells and H1299 + 5-Aza cells. Black circles denote DNA methylation sites. White circles denote unmethylated sites. **p < 0.01, ****p < 0.0001, NS: Not significant, and *p* < 0.05 were considered significant.

To explore why ALDH2 expression was reduced in LUAD tissues when compared to normal tissues, we assessed the basic expression of ALDH2 in different LUAD cell lines and normal bronchial epithelial (HBE) cells (Figures 2A, B). Following treatment with 5-Aza, a methyltransferase inhibitor, the expression level of ALDH2 was upregulated in H1299, H460, and HCC827 cells (Figures 2C, D). However, the level of ALDH2 in A549 and HBE cells was not changed. These findings indicate that ALDH2 may be regulated by DNA methylation in lung cancer cell lines. To study the DNA methylation of human ALDH2, we used MethPrimer (http://www.urogene.org/methprimer/) to predict the potential methylated CpG islands at this locus (22). One potential methylated CpG island (island 3) was the longest CpG island area in this gene (275 bp, at −264 to +10 bp), and this sequence is shown in Supplementary Figure 1A. We obtained genomic DNA from H1299 cells, which demonstrated the most significant increase in ALDH2 after 5-Aza treatment, and bisulfite-treated island 3 was amplified (Supplementary Figure 1B). QUMA (http://quma.cdb.riken.jp/top/) was used to analyze the methylation sites. The results indicate that an average of 78.18% of the sites in CpG island 3 was methylated (the black and white plots denote methylated sites and unmethylated sites, respectively; Figure 2E), and the methylation sites are almost demethylated after 5'AZA treatment (Figure 2F). These results further suggest that DNA methylation of the ALDH2 gene locus leads to loss of expression of ALDH2.

### ALDH2 Expression Is Upregulated After Knockdown of Either MBD4 or DNMT3A

**Figure 3:**
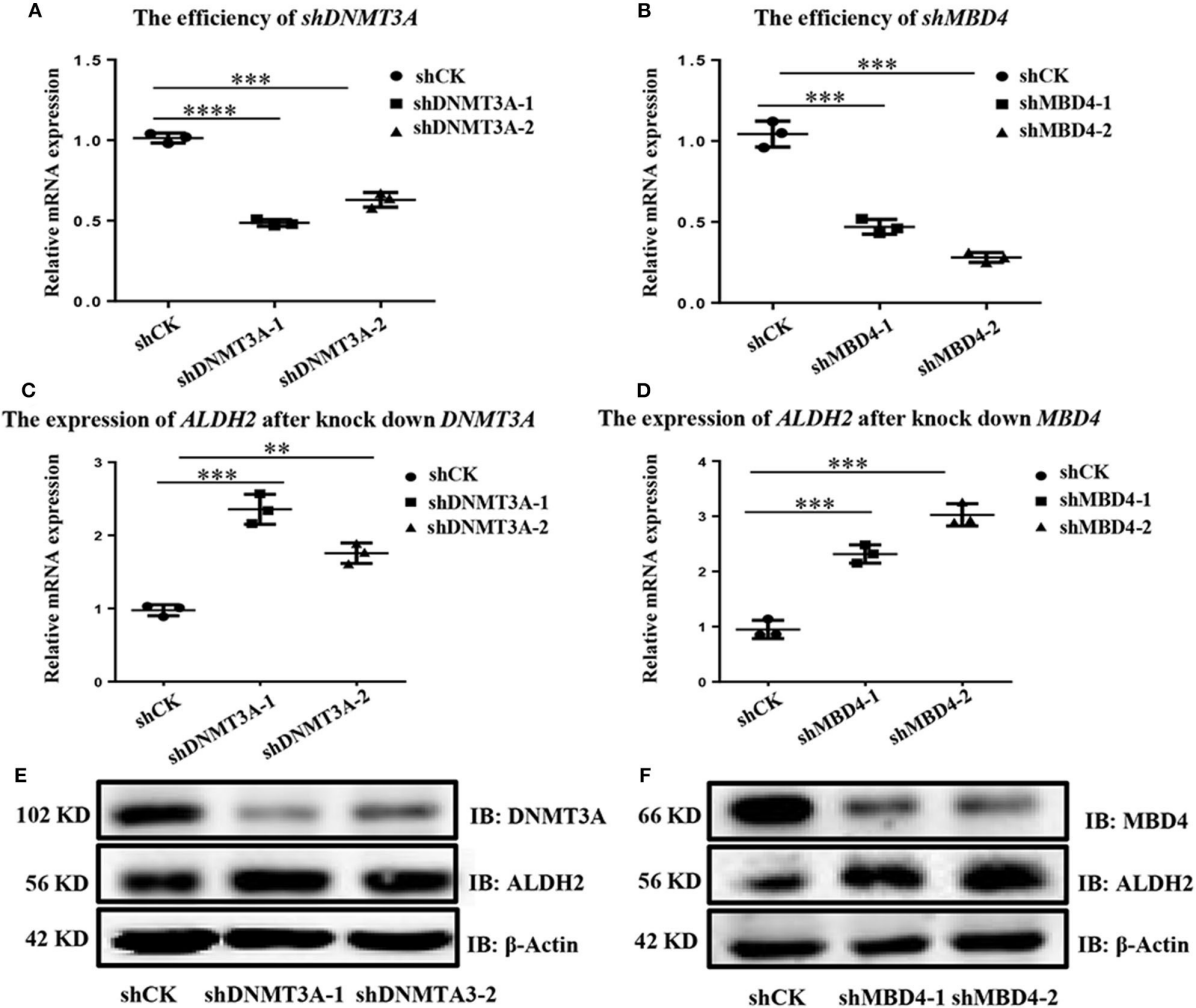
ALDH2 *expression* is upregulated after knockdown of either MBD4 or DNMT3A. RT-PCR showed the efficiency of shDNMT3A **(A)** and shMBD4 **(B)**. ALDH2 expression after knockdown of DNMT3A **(C)** and MBD4 **(D)**. **(E)** Western blot showed the efficiency of shDNMT3A and ALDH2 expression after knockdown of DNMT3A. **(F)** Western blot showed the efficiency of shMBD4 and ALDH2 expression after knockdown of MBD4. **p < 0.01, ***p < 0.001, ****p < 0.0001, and *p* < 0.05 were considered significant.

To find which DNA methyltransferases (DNMTs), demethylated transferases (TETs), and methylated CpG binding proteins (MBDs) play an important role in ALDH2 DNA methylation, we further analyzed the expression of ALDH2 in A549 cells, which displayed the highest expression of ALDH2, and in H1299 cells, which displayed the lowest expression of ALDH2 at the transcriptional level. As we expected, *DNMT1, DNMT3A*, and *DNMT3B* were all highly expressed in H1299 cells (Supplementary Figure 2A). The demethylated transferases *TET1, TET2*, and *TET3* were all downregulated in H1299 cells (Supplementary Figure 2B). While the *MBD1* and *MBD4* methylated CpG binding proteins were significantly upregulated in these cells when compared to their levels in A549 cells (Supplementary Figure 2C). This further supports that ALDH2 having a lower methylation level in A549 leads to increased ALDH2 expression. Samuel et al. (23) reported that the overexpression of DNMT3A promoted intestinal tumorigenesis. MBDs specifically recognize and bind to methylated DNA, and they can inhibit the assembly of chromosomes by binding to methylated DNA and recruiting other proteins to inhibit the transcription of target genes (24, 25). Therefore, we explored the effects of the knockdown of MBD4 and DNMT3A using short hairpin (sh) MBD4 and shDNMT3A RNAs (Figures 3A, B). After the knockdown of MBD4 and DNMT3A, ALDH2 was upregulated significantly not only at the mRNA level (Figures 3C, D) but also at the protein level (Figures 3E, F). These results further indicate that MBD4 and DNMT3A are crucial in the expression of ALDH2, and their heightened expression in H1299 cells leads to low levels of ALDH2.

### ALDH2 Expression Is Significantly Negatively Correlated to Cell Survival in *in vitro* Cell Culture Condition

**Figure 4:**
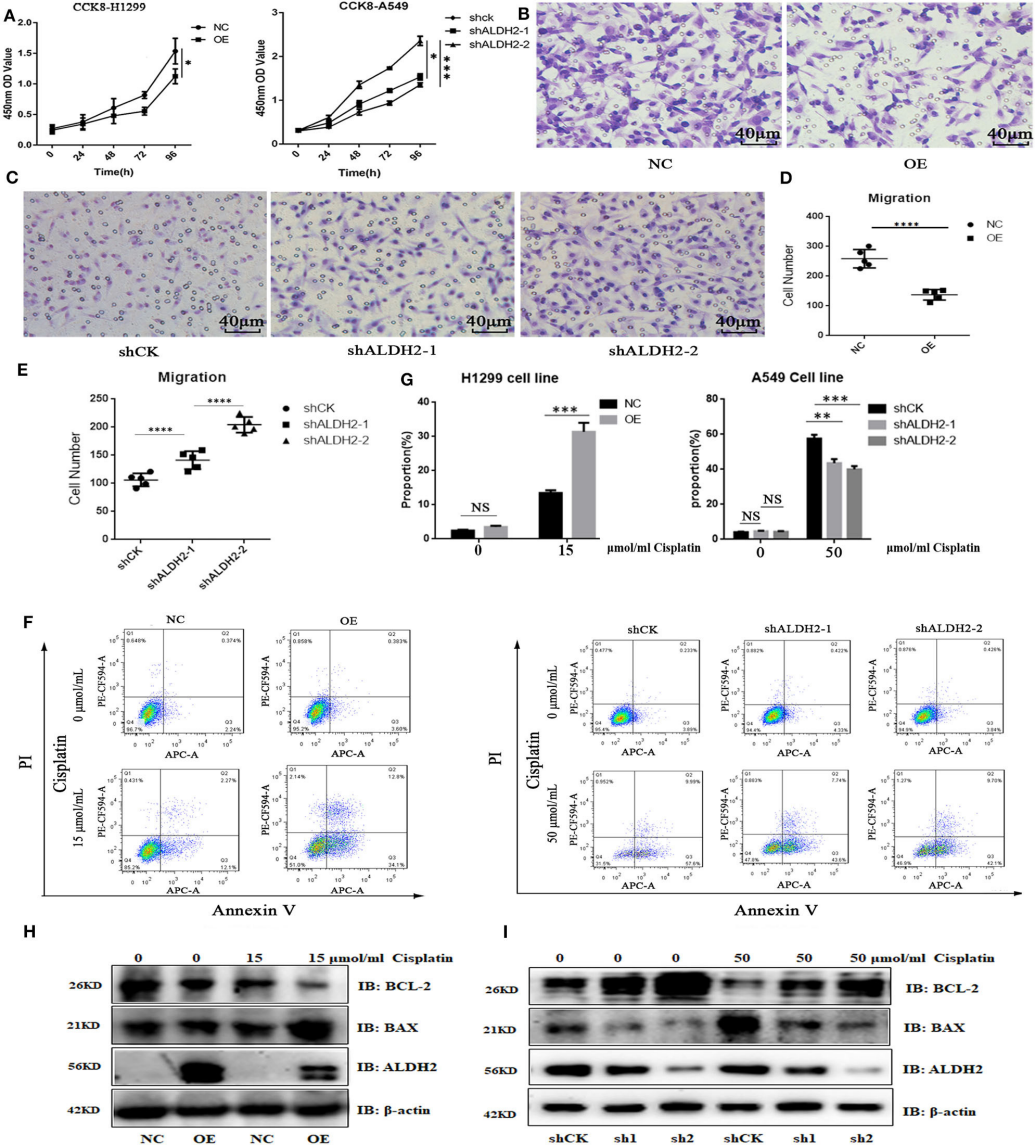
ALDH2 expression is significantly negatively correlated with cell survival *in vitro*. **(A)** Cell proliferation was detected using CCK-8. Transwell assays were used to test cell migration in H1299 **(B,D)** and A549 **(C,E)** cells. Fluorescence-activated cell sorting verified that the overexpression of ALDH2 promoted cell apoptosis in H1299 cells and that knockdown of ALDH2 inhibited cell apoptosis in A549 cells **(F,G)**. Western blot showed the expression of apoptosis proteins BAX and BCL2 in PLVX-Flag H1299 and PLVX-Flag-ALDH2 H1299 cells **(H)** and shCK A549 and shALDH2 A549 cells **(I)**. *p < 0.05, **p < 0.01, ***p < 0.001, ****p < 0.0001.

To prove the effects of ALDH2 on cell functions, we overexpressed ALDH2 in H1299 cells and knocked down ALDH2 expression in A549 cells. The efficiency of both procedures is presented in Supplementary Figures 3A, B. The CCK-8 viability assay showed that ALDH2 overexpression inhibited cell proliferation, whereas knockdown of ALDH2 promoted cell proliferation (Figure 4A). The transwell assay was used to assess whether ALDH2 affected cell migration. Overexpression of ALDH2 suppressed H1299 cell migration (Figures 4B, D), whereas knockdown of ALDH2 promoted A549 cell migration (Figures 4C, E). Cisplatin is a commonly used agent to induce apoptosis (26, 27). We further explored whether ALDH2 can induce apoptosis through cisplatin and found that overexpression of ALDH2 promoted cell apoptosis and that knockdown of ALDH2 inhibited apoptosis (Figures 4F, G). BAX and BCL-2 are important apoptosis protein markers (28), so we also did western bolt to further check the apoptosis. We found that pro-apoptosis protein BAX was elevated and that apoptosis suppressor BCL-2 was attenuated in the Flag-ALDH2 H1299 cell line. shALDH2 A549 cell lines showed the opposite situation (Figures 4H, I).

### ALDH2 Actively Represses MAPK Activation to Inhibit Cell Proliferation and Migration

**Figure 5:**
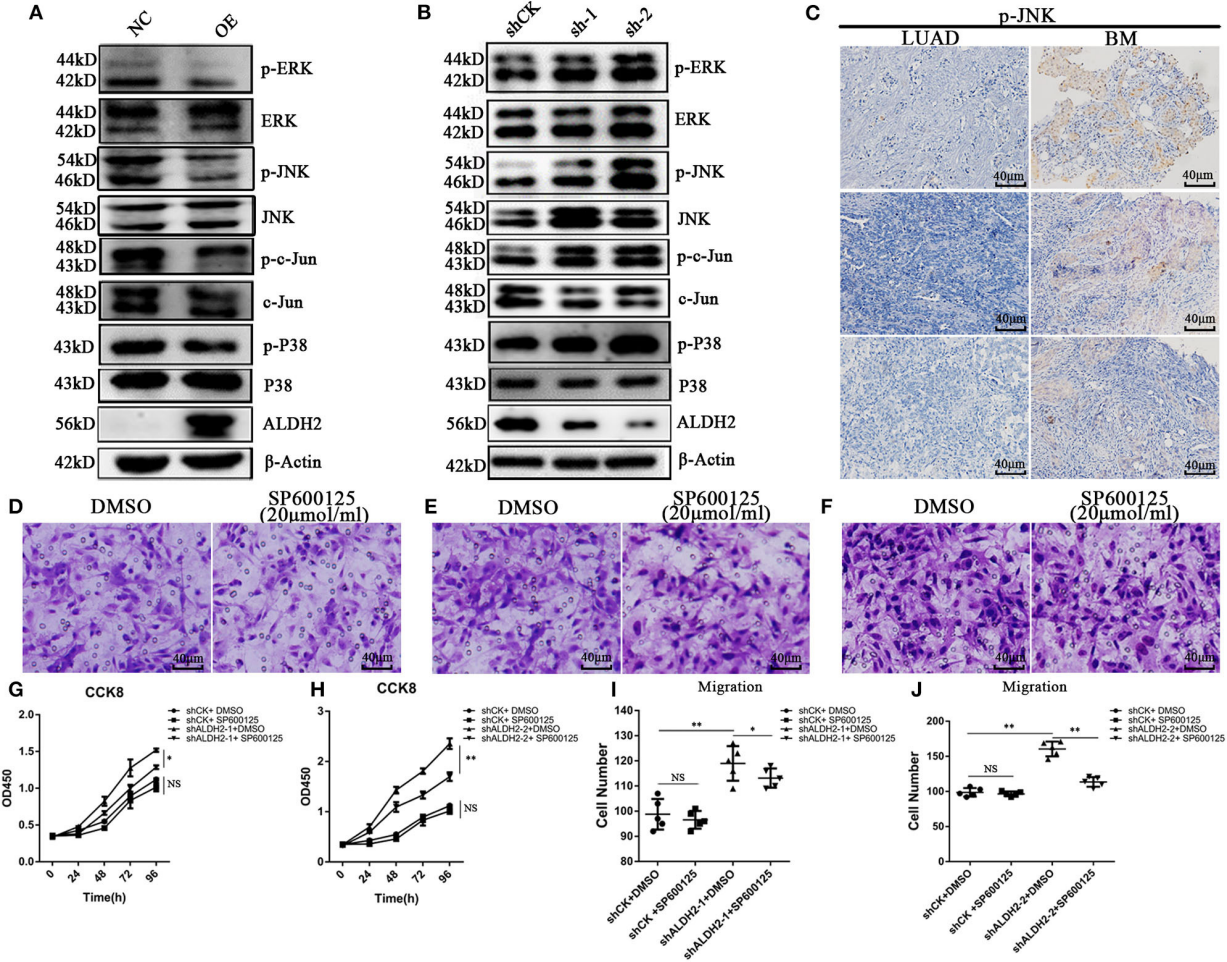
ALDH2 actively represses MAPK activation inhibiting cell proliferation and migration. **(A)** Phosphorylation of ERK1/2, and P38 and JNK/c-Jun modifications in PLVX-Flag-ALDH2 H1299 and PLVX-Flag H1299 cells. **(B)** Expression of phosphorylated ERK1/2, and P38 and JNK/c-Jun modifications in shCK A549 and shALDH2 A549 cells. **(C)** IHC was used to explore the expression of p-JNK in BM. Migration assay were performed with or without SP600125, a JNK inhibitor, in shCK **(D)**, shALDH2-1 **(E)**, and shALDH2-2 A549 **(F)** cells. Suppression of JNK could rescue cell proliferation **(G,H)**. **(I,J)** The statistical datas of migration assay treat with SP600125. *p < 0.05, **p < 0.01.

To demonstrate causality, we examined the MAPK signal pathway, which is related to the regulation of cell proliferation, migration, and apoptosis. Overexpression of ALDH2 inhibited phosphorylation of extracellular signal-related kinase (ERK)1/2, P38, and JNK/c-Jun modifications in H1299 cells (Figure 5A). p-JNK and p-P38 were activated in shALDH2 cell lines (Figure 5B). p-JNK was also more elevated in BM tissues than LUAD tissues (Figure 5C). SP600125, a highly efficient inhibitor of JNK, was added to inhibit the expression of JNK in shCK and shALDH2 A549 cells and then migration assay was preformed (Figures 5D-F). The statistical results showed that suppression of JNK could rescue cell proliferation (Figures 5G, H) and migration (Figures 5I, J) in shALDH2 A549 cell lines.

### ALDH2 Is Essential for EMT Progression via the Elevation of E-Cadherin and Attenuation of Vimentin Expression

**Figure 6:**
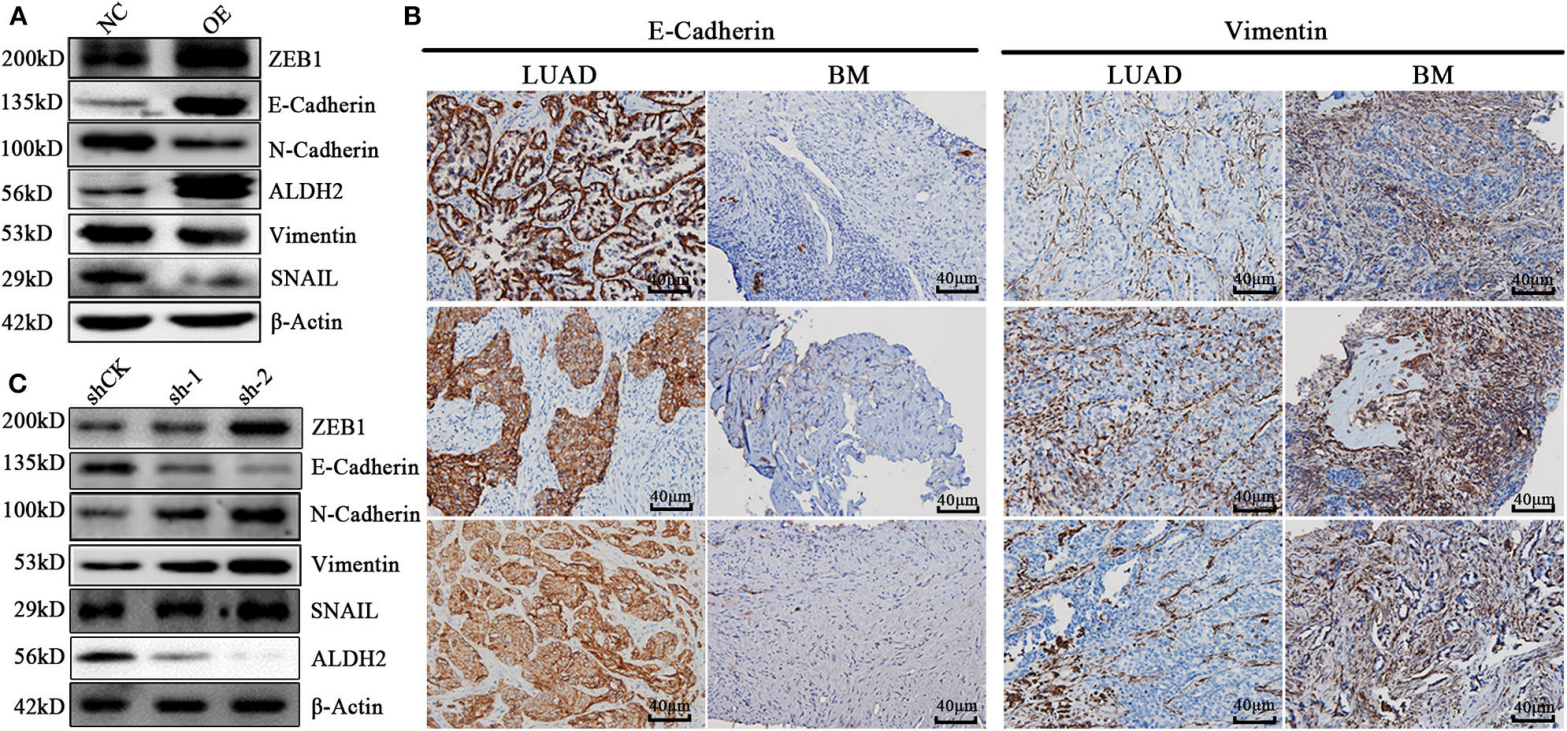
ALDH2 is essential for EMT progression through the elevation of E-cadherin level and attenuation of vimentin expression. **(A)** Expression levels of the EMT-related proteins ZEB1, E-cadherin, N-cadherin, vimentin, and SNAIL in PLVX-Flag-ALDH2 and PLVX-Flag H1299 cells. **(B)** IHC was used to explore the expression of E-cadherin and vimentin in BM. **(C)** Expression levels of the EMT-related proteins ZEB1, E-cadherin, N-cadherin, vimentin, and SNAIL in shCK A549 and shALDH2 A549 cells.

To explore the molecular mechanisms underlying the ALDH2-mediated attenuation of LUAD metastasis, we probed the relationship between ALDH2 and the EMT genes which are critical for tumor metastasis. The expression of the EMT-related genes encoding E-cadherin was upregulated in PLVX-Flag-ALDH2 H1299 cells, while the genes encoding N-cadherin, vimentin, and SNAIL were downregulated (Figure 6A). Meanwhile, E-cadherin was downregulated, while N-cadherin, ZEB1, and vimentin were upregulated in shALDH2 cell lines (Figure 6C). E-Cadherin is degraded, and vimentin (Figure 6B) is increased in BM tissues when compared to LUAD tissues. In the univariate analysis, AJCC; T stage, N stage, and M stage; and ALDH2, p-JNK, E-cadherin, and vimentin expression levels were all shown to be related to OS (Table 4). We went on to perform multivariate analysis on those variables shown to be significant in the univariate analysis. This analysis revealed that M stage (*p* = 0.003), ALDH2 (*p* = 0.008), and p-JNK (*p* = 0.027) expression levels were all independent prognostic factors for OS (Table 5).

**Table 4:** Univariate analysis for overall survival (OS).

**Variable**	**OS**		
	**HR**	**95% CI**	***P*-value**
**Age, years**			
<65	–		
≥65	1.876	0.846–4.160	0.122
**Gender**			
Male	–		
Female	0.974	0.430–2.207	0.950
**Smoker**			
Yes	–		
No	6.573	0.887–48.692	0.065
**T stage**			
T1+T2	–		
T3+T4	3.163	1.357–7.372	0.006*
**N stage**			
N0	–		
N1	3.038	1.330–6.936	0.008*
**M stage**			
M0	–		
M1	13.530	1.812–101.017	0.011*
**AJCC stage**			
I + II	–		
III + IV	37.01	0.648–2112.921	0.080
**ALDH2 expression**			
Low	–		
High	0.140	0.040–0.489	0.002*
**p-JNK expression**			
Positive	–		
Negative	5.194	1.759–15.337	0.003*
**E-cadherin expression**			
High	–		
Low	0.291	0.098–0.858	0.025*
**Vimentin expression**			
High	–		
Low	2.838	1.101–7.319	0.031*

* *p < 0.05 indicates a significant difference*.

**Table 5:** Multivariate analysis for OS.

**Variable**	**OS**		
	**HR**	**95% CI**	***P*-value**
M stage	4.364	1.641–11.603	0.003*
ALDH2	0.002	1.222–3.796	0.008*
p-JNK	3.480	1.150–10.529	0.027*

* *p < 0.05 indicates a significant difference*.

### ALDH2 Acts as a Tumor Suppressor by Suppressing Tumor Formation *in vivo*

**Figure 7:**
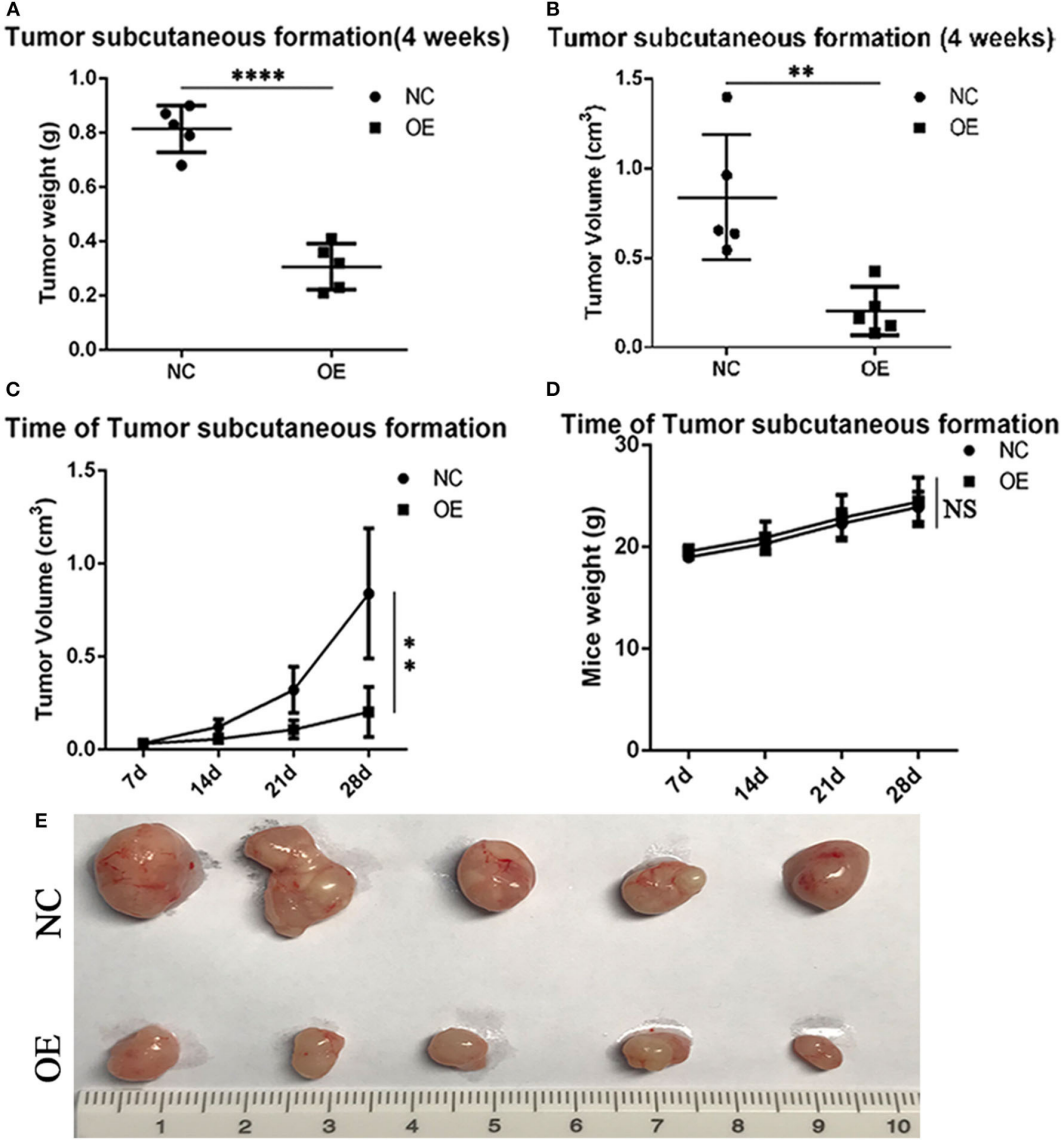
ALDH2 acts as a tumor suppressor by suppressing tumor formation *in vivo*. On the 28th-day post-injection with PLVX-Flag H1299 cells or PLVX-Flag-ALDH2 H1299 cells, the tumor weight **(A)** and tumor volume **(B)** were recorded (*p* < 0.05). **(C)** The size of the tumor in the overexpression and control group is shown (4 weeks). **(D)** The body weight of each of the two groups after injecting tumor cells (4 weeks). **(E)** The mice tumor samples. **p < 0.01, ****p < 0.0001, NS :Not significant.

*In vivo* experiments assessing subcutaneous tumor formation in mice showed that both tumor weight and tumor volume were greater in the PLVX-Flag H1299 mice than in the PLVX-Flag ALDH2 H1299 mice (*p* < 0.05; Figures 7A, B). And tumor size in the overexpression group was smaller than that in the control group (Figure 7C). We also demonstrated that there was no significant change in body weight (Figure 7D) for any of the animals in this study. The tumor size of the two groups is shown in Figure 7E.

## Discussion

Our data show that ALDH2 is differentially expressed in LUAD with BM and that ALDH2 expression is significantly negatively correlated with survival and BM. The high methylation rates for CpG island 3 in the promoter of ALDH2 result in the downregulation of ALDH2 in lung cancer cell lines. MBD4 and DNMT3A are involved in ALDH2 DNA methylation. Methylated ALDH2 greatly increases the probability of BM in lung cancer patients. Also, ALDH2 acts as a MAPK upstream to inhibit cell proliferation and migration and inhibits the EMT process by elevating E-cadherin and attenuating vimentin. Cell proliferation and migration are inhibited after adding JNK inhibitor SP600125. ALDH2 is also significantly related to clinical factors, including smoking; T, N, and M stages; AJCC; and p-JNK, E-cadherin and vimentin expression levels. In the multivariate analysis, M stage (*p* = 0.003), ALDH2 expression (*p* = 0.008), and p-JNK expression (*p* = 0.027) are independent prognostic factors for the OS of BM patients. Last, we find that ALDH2 acts as a tumor suppressor by suppressing tumor formation *in vivo*. These data identify potential biomarkers and therapeutic targets for LUAD, specifically for metastatic LUAD.

ALDH2 can catalyze the conversion of acetaldehyde to acetic acid. Lack of ALDH2 is related to elevated acetaldehyde concentration (18) and glucocorticoid post-alcohol consumption, which inhibits T-cell activation (29). This may increase the viability of tumor cells in circulation. Accumulation of glucocorticoids induces osteoporosis (30, 31) and destruction of osteoprotegerin (32), which may aggravate osteolytic destruction, osteoporosis, and BM. Thus, increasing the expression of ALDH2 is a potential therapeutic strategy for preventing and treating BM.

The activity of osteoblasts and osteoclasts is essential for BM, which is a multistep process (33, 34). Identification of altered epigenetic methylation patterns is a new and developing diagnostic direction (35). Nishikawa et al. identified DNMT3A-regulated osteoclastogenesis (36). They also found that expression of receptor activator of nuclear factor-kappa B ligand (RANKL) induced a metabolic shift by increasing the production of *S*-adenosylmethionine (SAM). DNMT3A can regulate epigenetic inhibition of anti-osteoclastogenic genes by SAM-mediated DNA methylation. In this study, we found that ALDH2 expression could be induced by inhibiting DNMT3A expression, which indicated that ALDH2 can be regulated by DNMT3A, greatly reducing the probability of BM in lung cancer patients. Thus, DNA methylation therapy may be essential in the treatment of BM.

BM is a multistep process that results from the complex interaction of tumor cells and osteoclasts. Osteolysis leads to the release of bone-derived growth factors, which bind to the receptors on the cancer cell's surface, activating autophosphorylation and the MAPK pathway (8, 37). Yang et al. found that constitutive activation of P38 MAPK in cancer cells contributed to osteolytic damage in patients with multiple myelomas (38). Sun et al. found that ALDH2 detoxifies 4-hydroxy-2-nonenal to elicit a cytosolic response through the JNK/p53 pathway (39). Xu et al. (40) demonstrated that mitochondrial ALDH overexpression attenuates hyperoxia-induced cell death in lung epithelial cells through reduction of reactive oxygen species and activation of ERK/MAPK cell survival signaling pathways. In this study, we upregulated ALDH2 and observed decreased phosphorylation of proteins involved in MAPK, which may inhibit LUAD BM.

In future studies, we will detect the DNA methylation level of ALDH2 in the human lung cancer samples and establish a BM model to further analyze the effects and mechanisms of ALDH2 on BM in lung cancer and EMT *in vivo*.

In summary, we demonstrate, for the first time, that DNMT3A and MBD4 attenuate ALDH2 expression and promote LUAD BM in a MAPK-dependent manner using human LUAD BM tissues. These data identify potential biomarkers and therapeutic targets for LUAD, specifically for LUAD with BM.

## Data Availability Statement

The datasets analyzed for this study can be found in TCGA database (https://portal.gdc.cancer.gov/) and Kaplan–Meier plotter (http://kmplot.com).

## Ethics Statement

The studies involving human participants were reviewed and approved by Ethics Committee of Shanghai Sixth People's Hospital. The patients/participants provided their written informed consent to participate in this study. The animal study was reviewed and approved by the Animal Ethics Committee of Shanghai Jiao Tong University affiliated Sixth People's Hospital. Written informed consent was obtained from the individual(s) for the publication of any potentially identifiable images or data included in this article.

## Author Contributions

MY: development or design of study methodology, programming, application of statistical, mathematical, computational methods, and writing. AW: data curation and project administration. CL, JS, and XL: management of research plans and execution of experiments. ZW, GYa, YZ, and SW: collection of clinical samples and investigation. GYi and HC: validation of experimental details and research outputs. DL and RL: provision of study materials, patients, laboratory samples, and instrumentation tools. HZ and BL: formulation of overarching research goals and aims, supervision, and acquisition of funding. All authors contributed to the article and approved the submitted version.

## Conflict of Interest

BL is a co-founder of Biotheus Inc and chairman of its scientific advisory board. The remaining authors declare that the research was conducted in the absence of any commercial or financial relationships that could be construed as a potential conflict of interest.

## Supplementary Material

Supplementary Figure 1 CpG island 3 sequence and Bisulfite-PCR sequencing chromatograms. **(A)** CpG island 3 sequences, **(B)** bisulfite-PCR sequencing chromatograms.

Supplementary Figure 2 The expression levels of Methyltransferase, demethylated transferases, Methylated CpG binding proteins are upregulated in A549 and H1299 cell lines. The expression of Methyltransferase **(A)** *DNMT1, DNMT3A*, and *DNMT3B*; demethylated transferases **(B)** *TET1, TET2*, and *TET3*; methylated CpG binding proteins **(C)** *MBD1, MBD2*, and *MBD4* in A549 and H1299 cell lines. ^****^*p* < 0.0001.

Supplementary Figure 3 Expression of ALDH2 after overexpression or knockdown with shRNA constructs. ALDH2 overexpression in H1299 cells and knockdown efficiency in A549 cells analyzed by RT-PCR **(A)** and Western blot **(B)**.
