# Supplementary material for: Methylation-Induced Silencing of ALDH2 Facilitates Lung Adenocarcinoma Bone Metastasis by Activating the MAPK Pathway
Source: Front Oncol. 2020 Jul 30;10:1141. doi: 10.3389/fonc.2020.01141 (PMC7406638; doi:10.3389/fonc.2020.01141)

A ALDH2 CpG island 3 sequence: -264bp to +10bp

GGCACACATTGGGGGCTCAACCAAGGCGAGCTGCGTTCGCGGGCCGGGTCTTTCCGCACAGGCGGA
GGGCGGTGGCGGGCGCGGAGGCGTCGCGCGAGCCAGGGGGCAGCCACGGGCCGGGGGTACCTAGCG
CCACCCGCTTCGCTTGCATCAGCTGCGCGCCCCATCCCGAGGAATGGTAGAGGCAGCCCCGCCCCCGG
CCCGCCCCCGCCTTTCCATTGGCTGCCGCGCGGGGCGGGGAGCGGGGTCGGCTCAGTGGCCCTGAGA
CCCTAGC

B

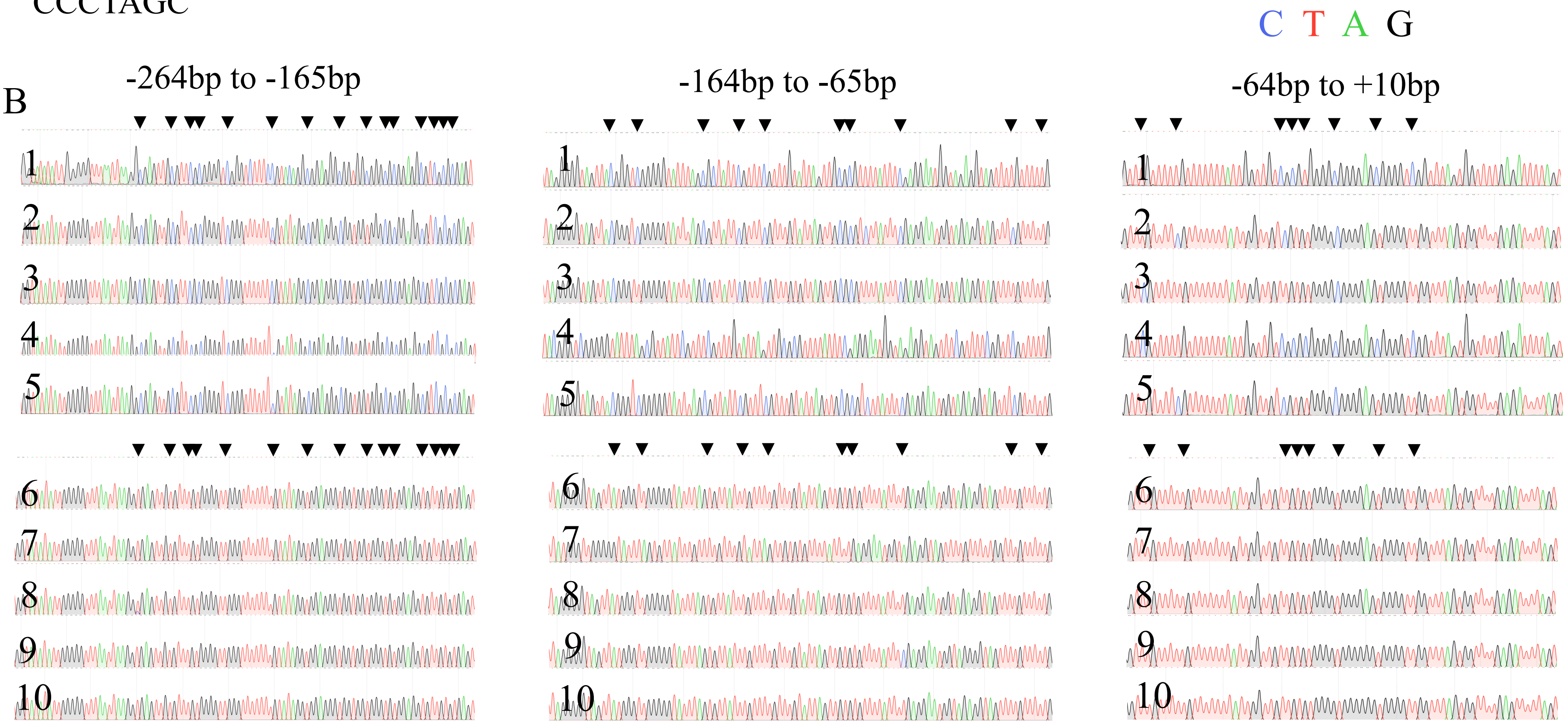

Supplement: Supplementary Figure 1 — CpG island 3 sequence and Bisulfite-PCR sequencing chromatograms. (A) CpG island 3 sequences, (B) bisulfite-PCR sequencing chromatograms. [file Image_1.pdf]
