# Supplementary figures and images for: Methylation-Induced Silencing of ALDH2 Facilitates Lung Adenocarcinoma Bone Metastasis by Activating the MAPK Pathway
Source: Front Oncol. 2020 Jul 30;10:1141. doi: 10.3389/fonc.2020.01141 (PMC7406638; doi:10.3389/fonc.2020.01141)

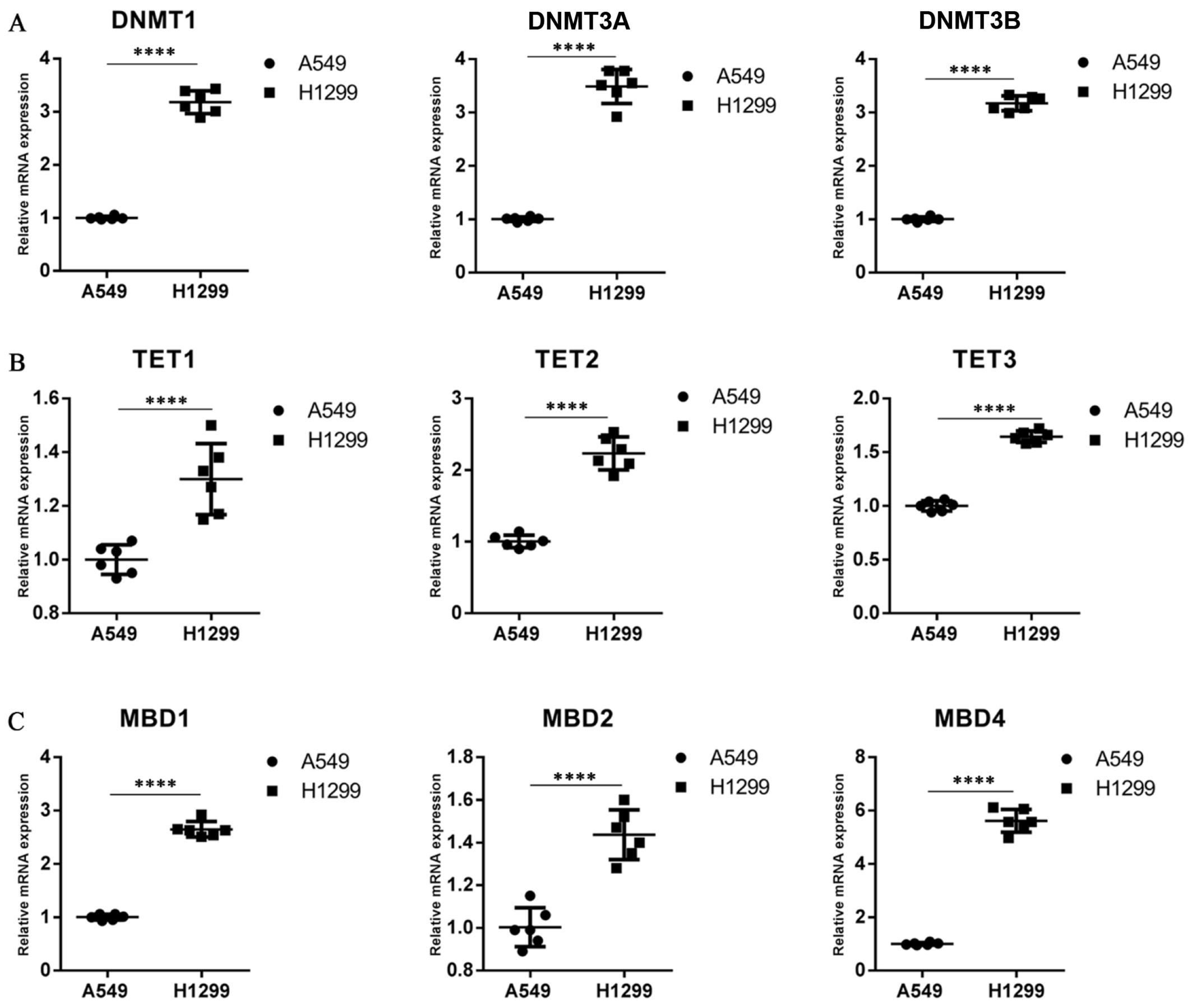

Supplement: Supplementary Figure 2 — The expression levels of Methyltransferase, demethylated transferases, Methylated CpG binding proteins are upregulated in A549 and H1299 cell lines. The expression of Methyltransferase (A) DNMT1, DNMT3A, and DNMT3B; demethylated transferases (B) TET1, TET2, and TET3; methylated CpG binding proteins (C) MBD1, MBD2, and MBD4 in A549 and H1299 cell lines. ****p < 0.0001. [file Image_2.TIF]

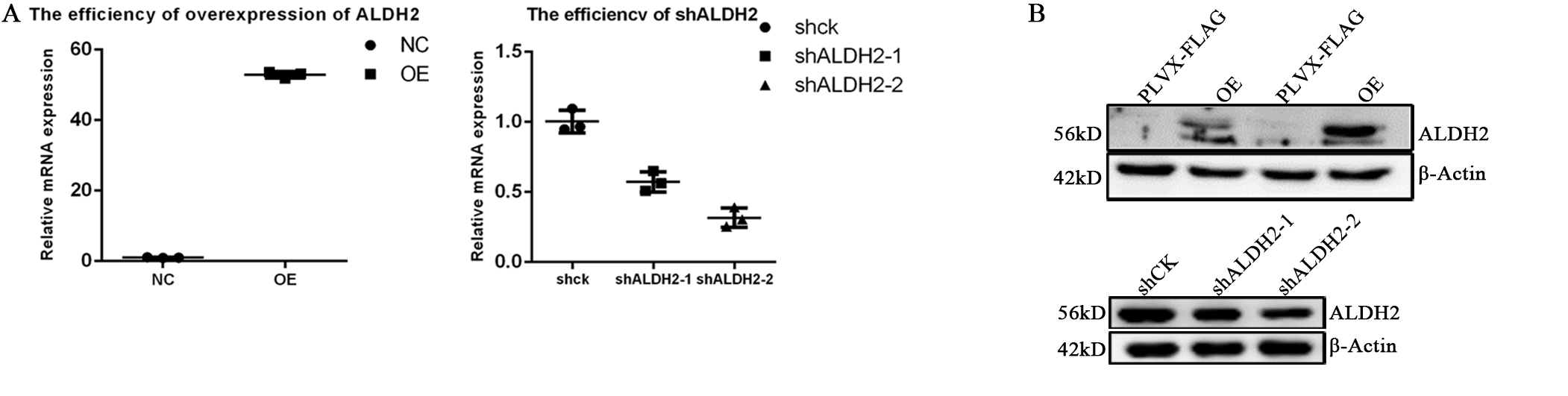

Supplement: Supplementary Figure 3 — Expression of ALDH2 after overexpression or knockdown with shRNA constructs. ALDH2 overexpression in H1299 cells and knockdown efficiency in A549 cells analyzed by RT-PCR (A) and Western blot (B). [file Image_3.TIF]
